# Supplementary material for: Homeobox A4 suppresses vascular remodeling by repressing YAP/TEAD transcriptional activity
Source: EMBO Rep. 2020 Mar 9;21(4):e48389. doi: 10.15252/embr.201948389 (PMC7132199; doi:10.15252/embr.201948389)
Supplement: Supplementary file 1 — Appendix [file EMBR-21-e48389-s001.pdf]

Appendix for:

Homeobox A4 Suppresses Vascular Remodeling as a Novel Regulator of YAP/TEAD Transcriptional Activity

Masahiro Kimura, Takahiro Horie, Osamu Baba, Yuya Ide, Shuhei Tsuji, Randolph Ruiz Rodriguez, Toshimitsu Watanabe, Tomohiro Yamasaki, Chiharu Otani, Sijia Xu, Yui Miyasaka, Yasuhiro Nakashima, Takeshi Kimura, Koh Ono

Table of contents

Appendix Figure S1 - Identification of novel modulators of YAP/TEAD transcriptional activity using pooled shRNA screen..2

Appendix Figure S2 - HOXA4 attenuated the expression of YAP/TEAD target genes........3

Appendix Figure S3 - Gene expression levels of canonical Hippo pathway kinases were unaffected by HOXA4...4

Appendix Figure S4 - Expression of TEAD family genes..5

Appendix Figure S5 - Generation of Hoxa4 deficient mice...6

Appendix Figure S6 – Characteristics of Hoxa4-deficient mice...7

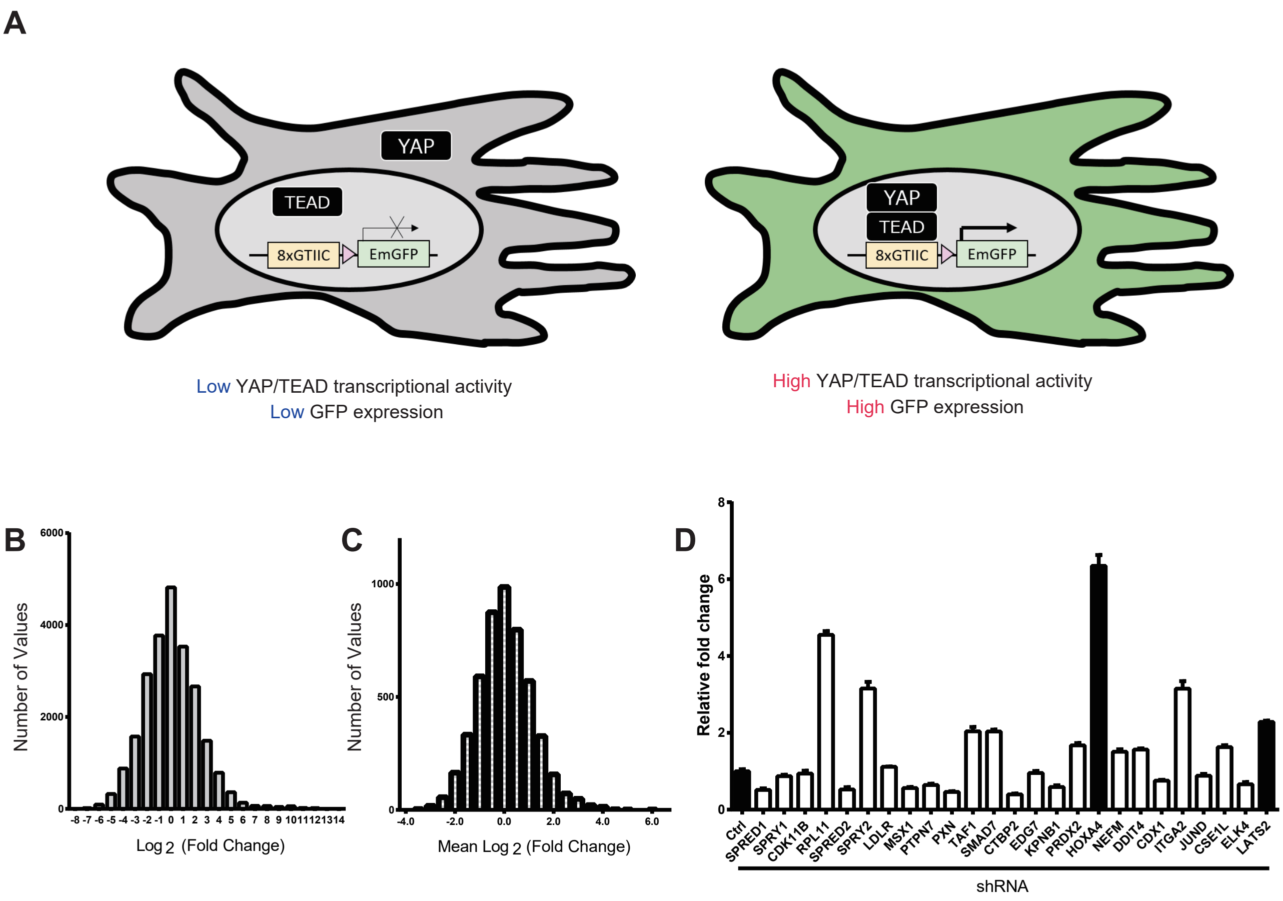

Appendix Figure S1 - Identification of novel modulators of YAP/TEAD transcriptional activity using pooled shRNA screen.

A, Schematic drawing of 8×GTIIC-EmGFP 293T cell line carrying eight TEAD-binding sequences upstream of a minimal promoter and EmGFP.

B and **C**, Distribution of read abundance per shRNA (B) or average read abundance of shRNA targeting each gene (C) followed an approximately Gaussian curve.

D, Validation of YAP/TEAD transactivation by knockdown of candidate genes using 8xGTIIC luciferase assay (n=3). Data are presented as mean ± SEM.

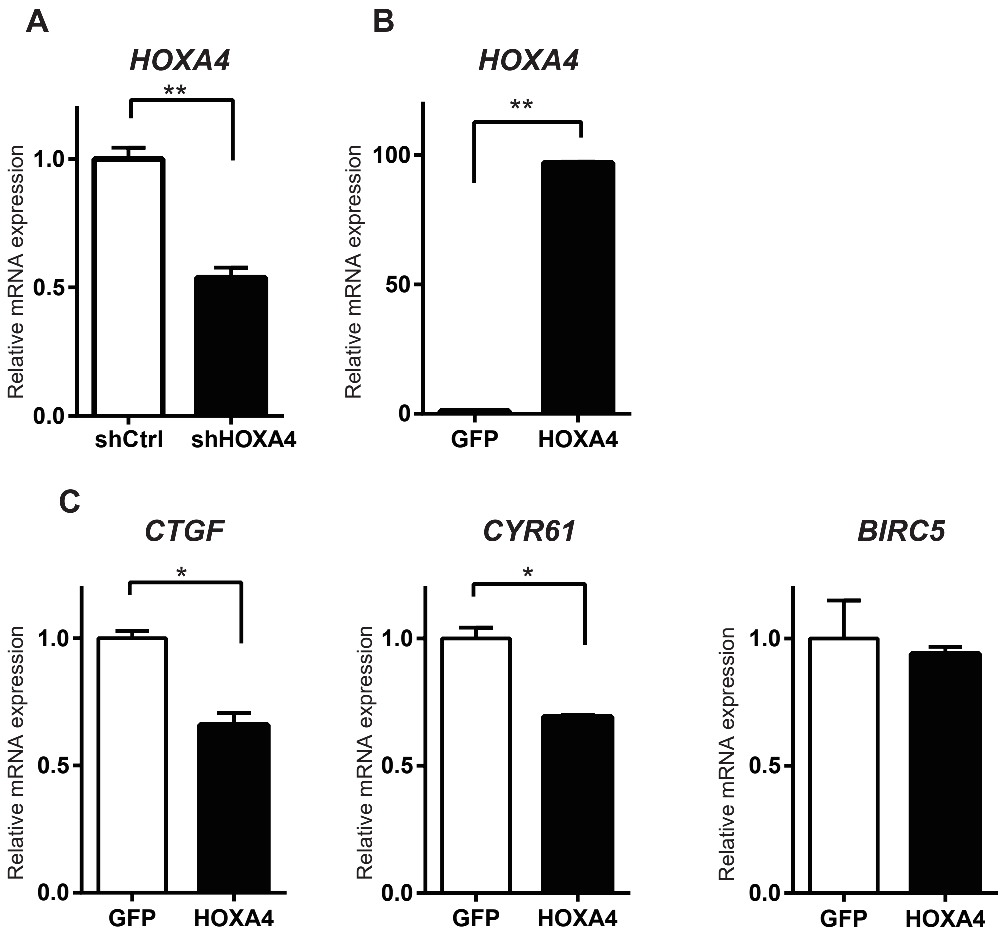

Appendix Figure S2 - HOXA4 attenuated the expression of YAP/TEAD target genes.

A, Knockdown efficacy of shHXOA4 vector in HEK 293T cells assessed by quantitative real-time PCR (n=3).

B and **C**, Quantitative real-time PCR analysis of HOXA4 (B) and YAP/TEAD target gene expression (C) in HEK 293T cells transfected with HOXA4 expression vector (n=3). All data are presented as mean ± SEM.

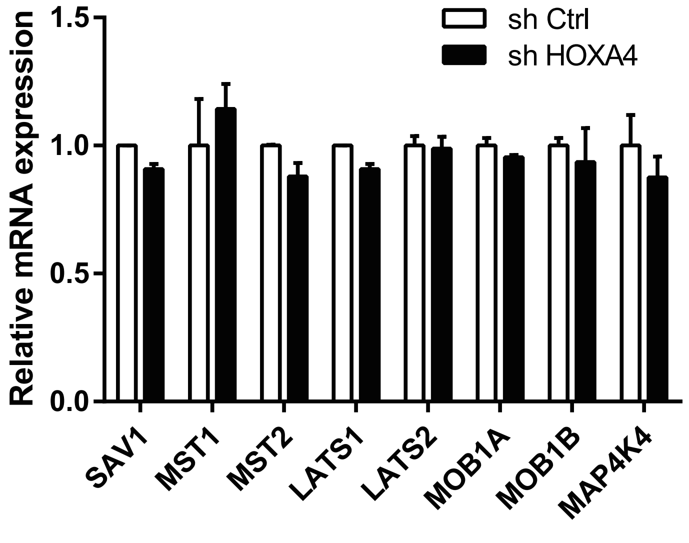

Appendix Figure S3 - Gene expression levels of canonical Hippo pathway kinases were unaffected by HOXA4.

Quantitative real-time PCR analysis of core Hippo pathway kinases in HEK 293T cells transfected with shHOXA4 (n=3). Data are presented as mean ± SEM.

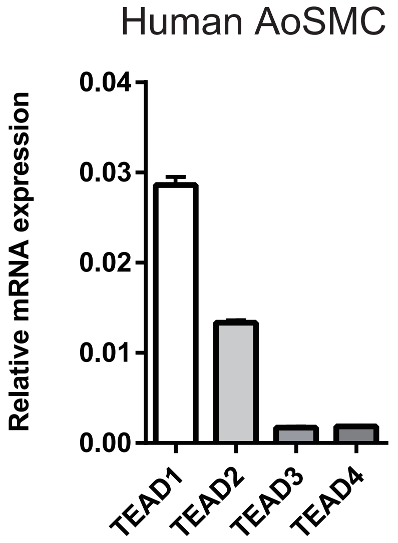

Appendix Figure S4 - Expression of TEAD family genes.

Quantitative real-time PCR analysis of TEAD family genes in human vascular smooth muscle cells (n=3). Data are presented as mean ± SEM.

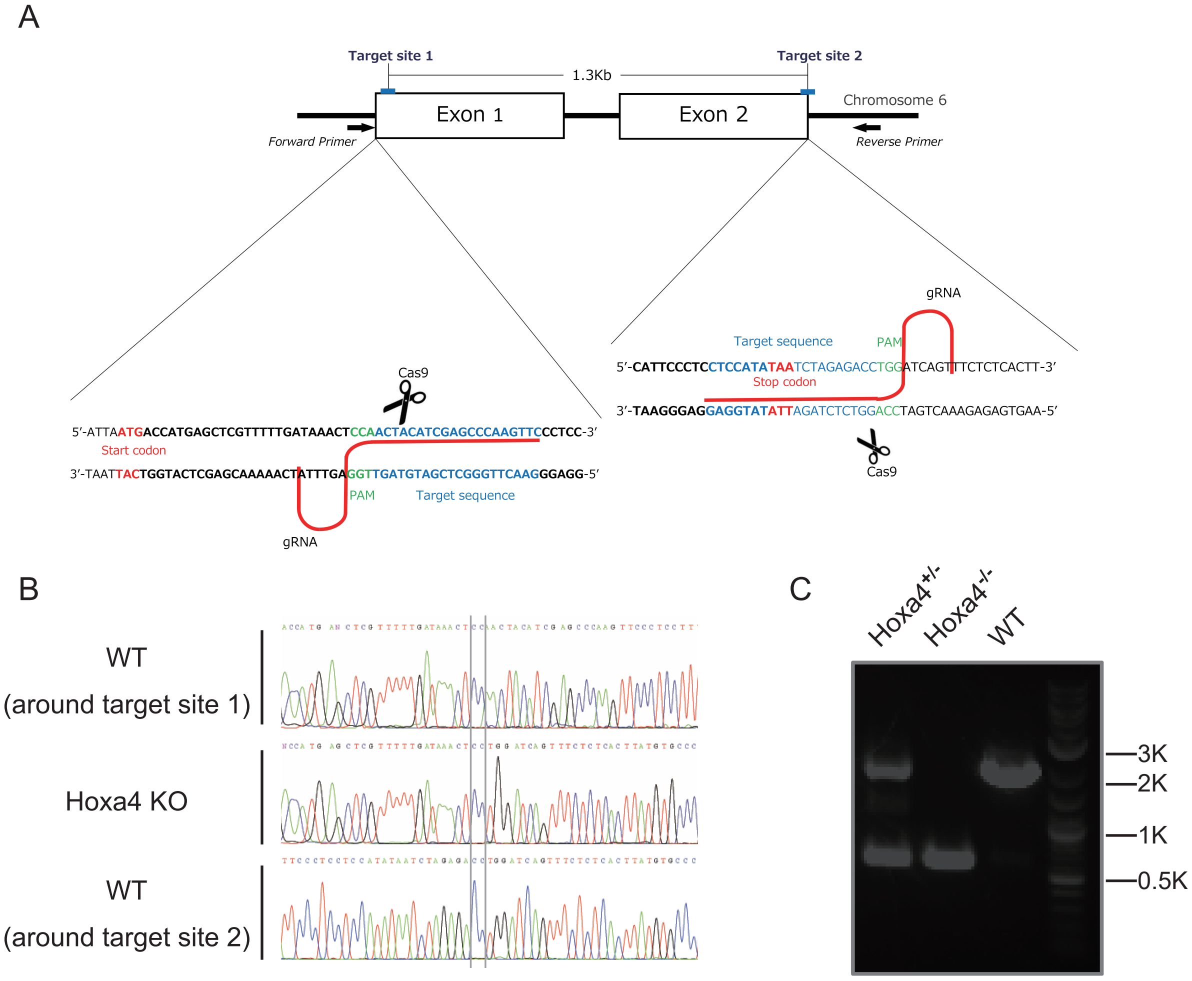

Appendix Figure S5 - Generation of Hoxa4 deficient mice.

A, Strategy to generate Hoxa4 KO mice.

B, DNA sequence around the target site of Cas9 cleavage in the knockout allele.

C, PCR analysis of mouse tail genome.

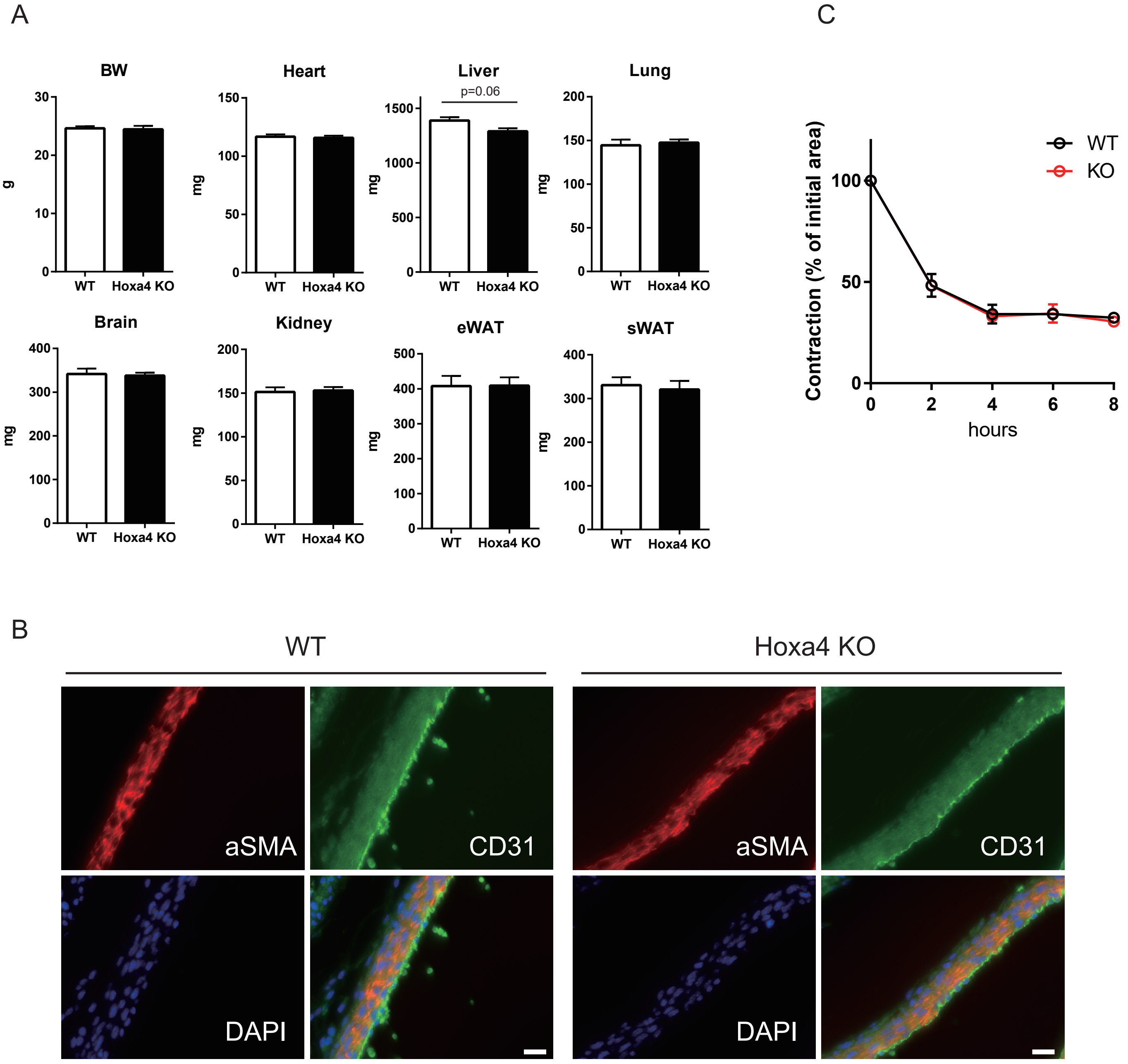

Appendix Figure S6 – Characteristics of Hoxa4-deficient mice.

A, Organ weight of 9-week-old male mice (WT: n=6, Hoxa4: n=8). Data are presented mean ± SEM and analyzed by unpaired two-tailed Mann–Whitney test. BW; body weight, eWAT; epididymal white adipose tissue, sWAT; subcutaneous white adipose tissue.

B, Representative immunofluorescent staining images for α-SMA and CD31 on dorsal aorta of embryonic day 18.5 (E18.5). Bars indicate 20 μm.

C, Collagen gel contraction assay of primary vascular smooth muscle cells harvested from 8-week-old male mice (n=3). Data are presented as mean ± SEM.
