# Supplementary material for: Homeobox A4 suppresses vascular remodeling by repressing YAP/TEAD transcriptional activity
Source: EMBO Rep. 2020 Mar 9;21(4):e48389. doi: 10.15252/embr.201948389 (PMC7132199; doi:10.15252/embr.201948389)
Supplement: Supplementary file 2 — Expanded View Figures PDF [file EMBR-21-e48389-s002.pdf]

Expanded View Figures

Figure EV1. HOXA4 negatively regulates YAP and TAZ-mediated TEAD transcriptional activity.

A Quantitative real-time PCR analysis of *HOXA4*, *YAP*, *CTGF*, and *CYR61* in HEK 293T cells with overexpression of HOXA4 and WT YAP. **$P < 0.01$ versus YAP overexpression with LacZ, by ANOVA with Sidak's correction. Data are presented as mean ± SEM of three independent experiments.

B Quantitative real-time PCR analysis of *HOXA4*, *YAP*, *CTGF*, and *CYR61* in HEK 293T cells with knockdown of endogenous HOXA4 and overexpression of WT YAP. *$P < 0.05$, **$P < 0.01$ versus YAP overexpression with shCtrl, by ANOVA with Sidak's correction. Data are presented as mean ± SEM of three independent experiments.

C Quantitative real-time PCR analysis of *CTGF* and *CYR61* in HEK 293T cells with knockdown or overexpression of HOXA4 and overexpression of TAZ. **$P < 0.01$ versus TAZ overexpression, by ANOVA with Sidak's correction. Data are presented as mean ± SEM of three independent experiments.

D Fluorescent images of HEK 293T cells transfected with GFP-HOXA4 fusion protein under different cell densities. Scale bars indicate 50 μm.

E Representative immunofluorescent staining images for YAP, GFP, and DAPI on HEK 293T cells transfected with GFP-HOXA4 fusion protein under different cell densities. Scale bars indicate 50 μm.

© 2020 The Authors

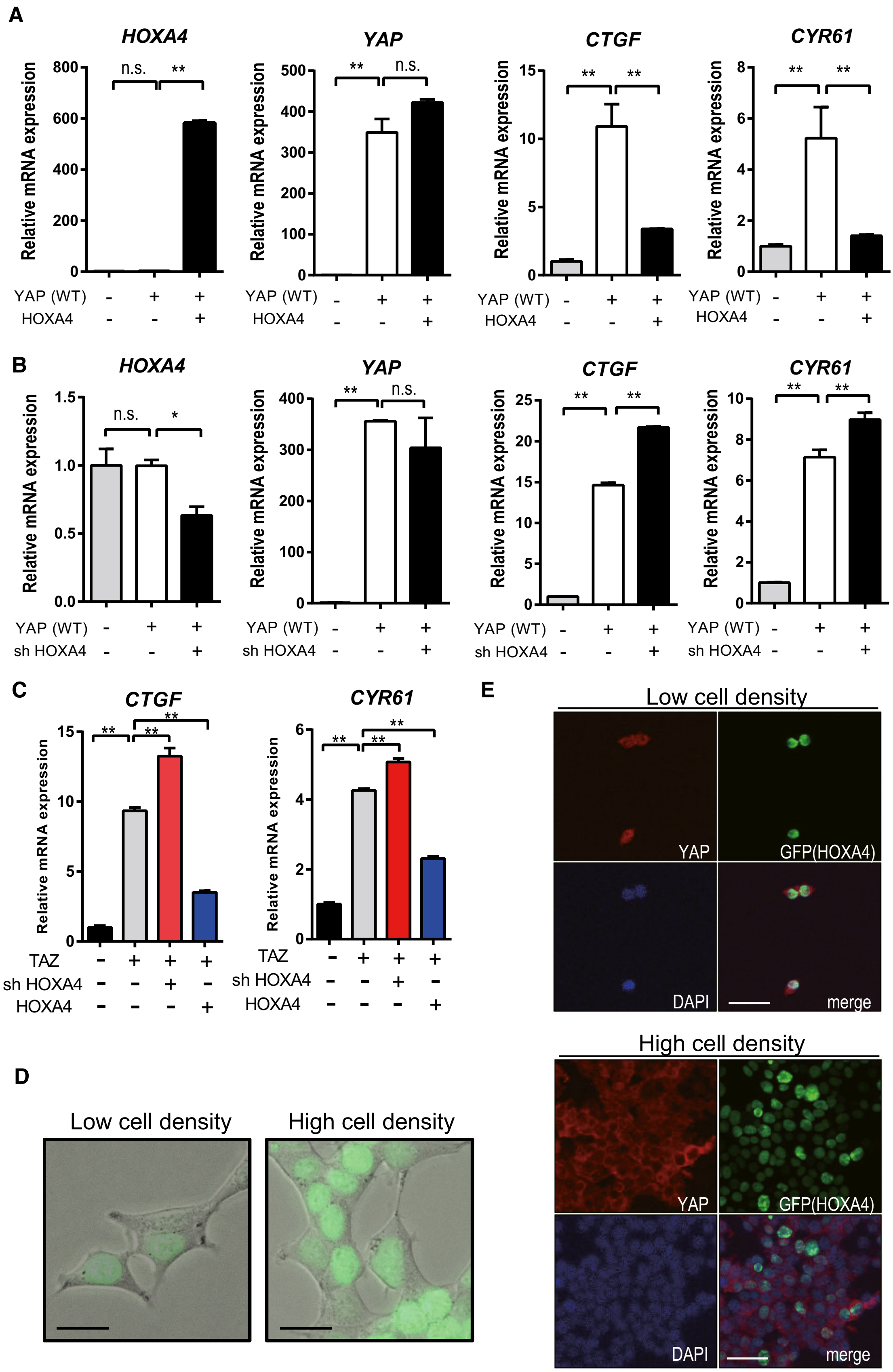

Figure EV1.

© 2020 The Authors

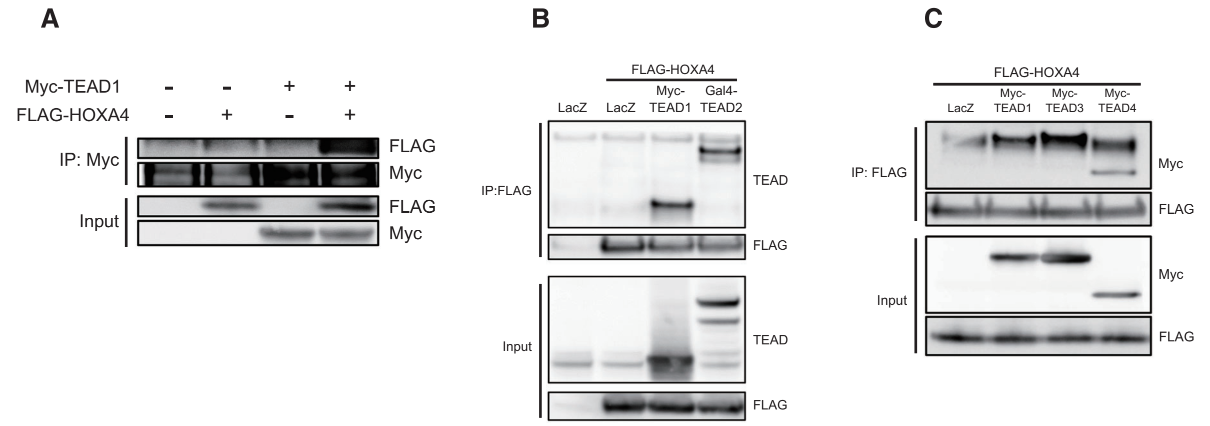

Figure EV2. HOXA4 interacts with TEADs in HEK 293T cells.

A Specific binding between TEAD1 and HOXA4. HEK 293 cells were transfected with Myc-TEAD1 and FLAG-HOXA4. The specificity of binding was confirmed by precipitation with anti-Myc antibody and further immunoblotting with anti-FLAG antibody.

B, C Co-IP experiments detecting the interaction between HOXA4 and all isoforms of TEADs.

Data information: All experiments above were repeated at least twice.

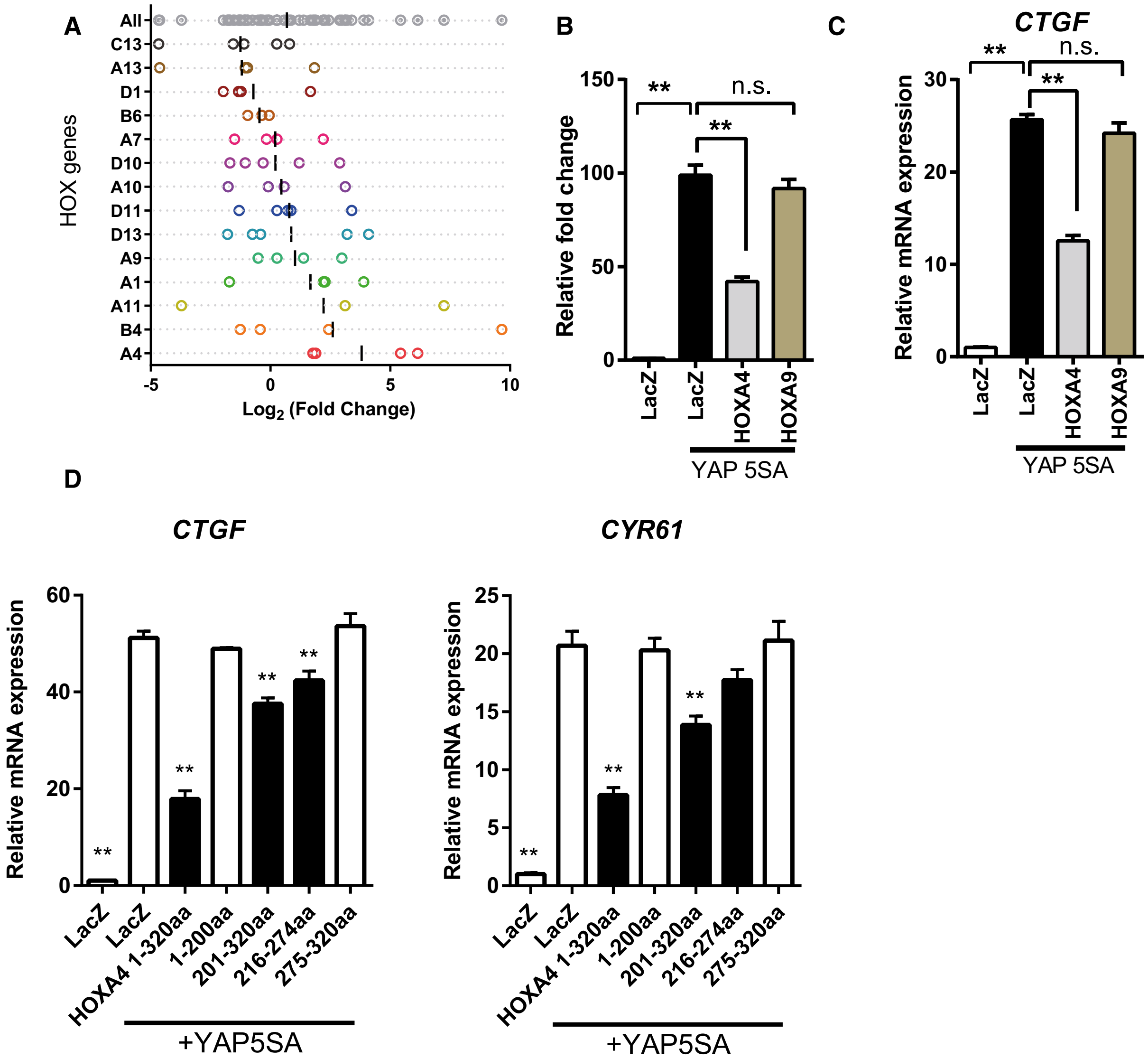

Figure EV3. Suppression of YAP/TEAD transcriptional activity is specific for HOXA4.

A Distribution of the frequency of each shRNA targeting HOX family genes in shRNA screen. Log2 (fold change) of each shRNA abundance in the "GFP high" pool compared with the "GFP low" pool is shown. Each dot represents different shRNAs, and vertical solid line represents the mean value. Three to five shRNAs against each HOX gene are contained in the library.

B, C Luciferase reporter assay using 8xGTIIC-luciferase (B) and quantitative real-time PCR of *CTGF* (C) in HEK 293T cells with overexpression of YAP-5SA and HOXA4 or HOXA9.

D Quantitative real-time PCR analysis of *CTGF* and *CYR61* in HEK 293T cells with overexpression of YAP-5SA and truncated mutants of HOXA4.

Data information: All data except for (A) are presented as mean ± SEM; three biological repeats. $^{**}P < 0.01$ versus LacZ with YAP-5SA, by ANOVA with Sidak's correction.

 © 2020 The Authors

Figure EV4. Cellular function of HOXA4 in vascular smooth muscle cells.

A FANTOM5 CAGE data showing higher expression levels of HOXA4 in human VSMCs.

B Representative immunofluorescent staining images for α-SMA and HOXA4 on human aortic samples. Scale bars indicate 20 μm.

C Expression of *Hoxa4* and markers of VSMC proliferation and contractile phenotype in mouse primary aortic smooth muscle cells stimulated with PDGF-BB. All data are presented as mean ± SEM of three independent experiments. $^{*}P < 0.05$, $^{**}P < 0.01$, by unpaired two-tailed Student's *t*-test.

D Western blotting analysis of cytosolic and nuclear protein levels of HOXA4, YAP, TAZ, and TEAD1 in human VSMCs.

E Immunocytochemistry of human VSMCs with anti-HOXA4 antibody. Scale bars indicate 50 μm.

F, G Representative images of BrdU incorporation assay in human VSMCs transduced with the indicated genes (related to Fig 6H and J). Scale bars indicate 200 μm.

© 2020 The Authors

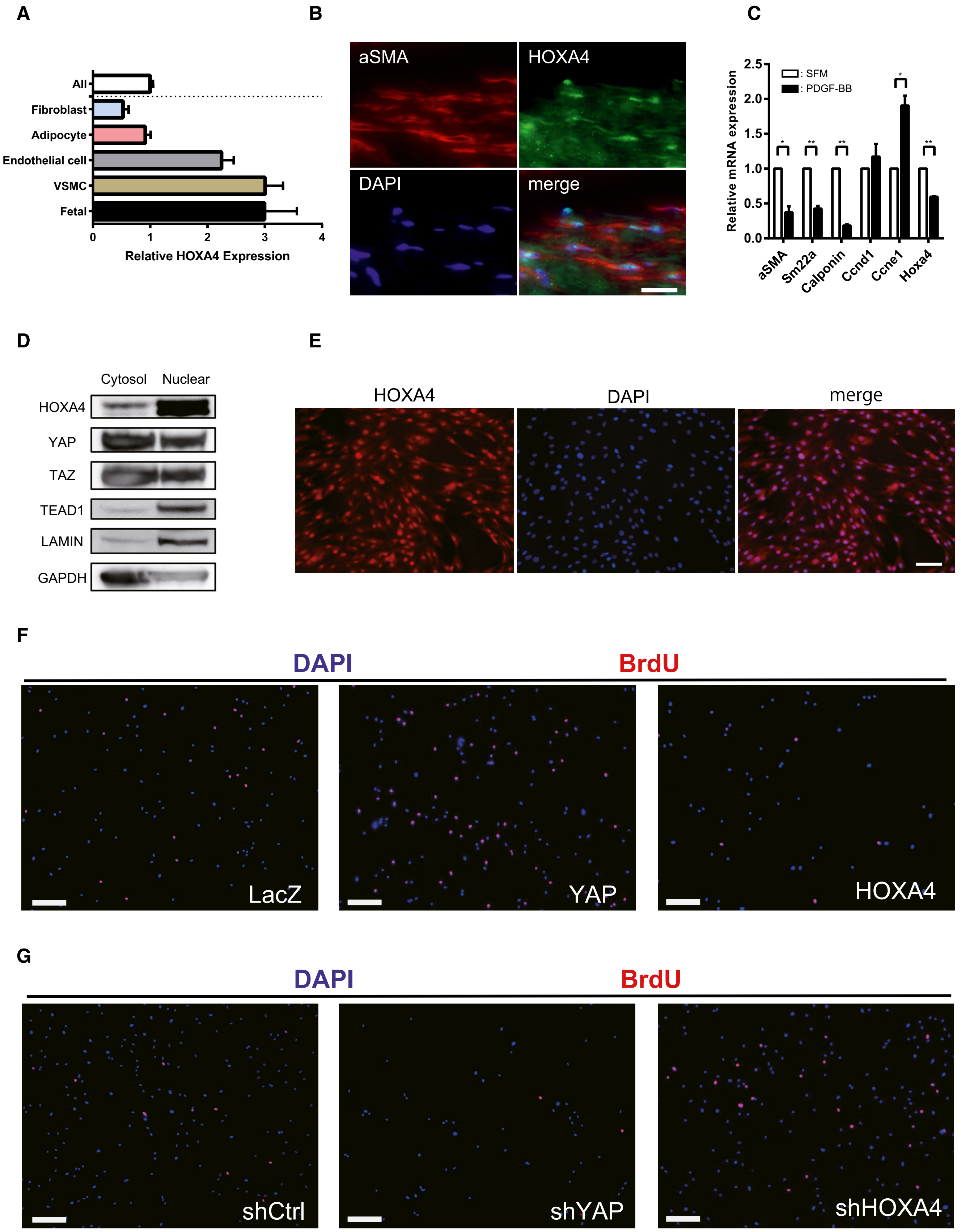

Figure EV4.

 © 2020 The Authors

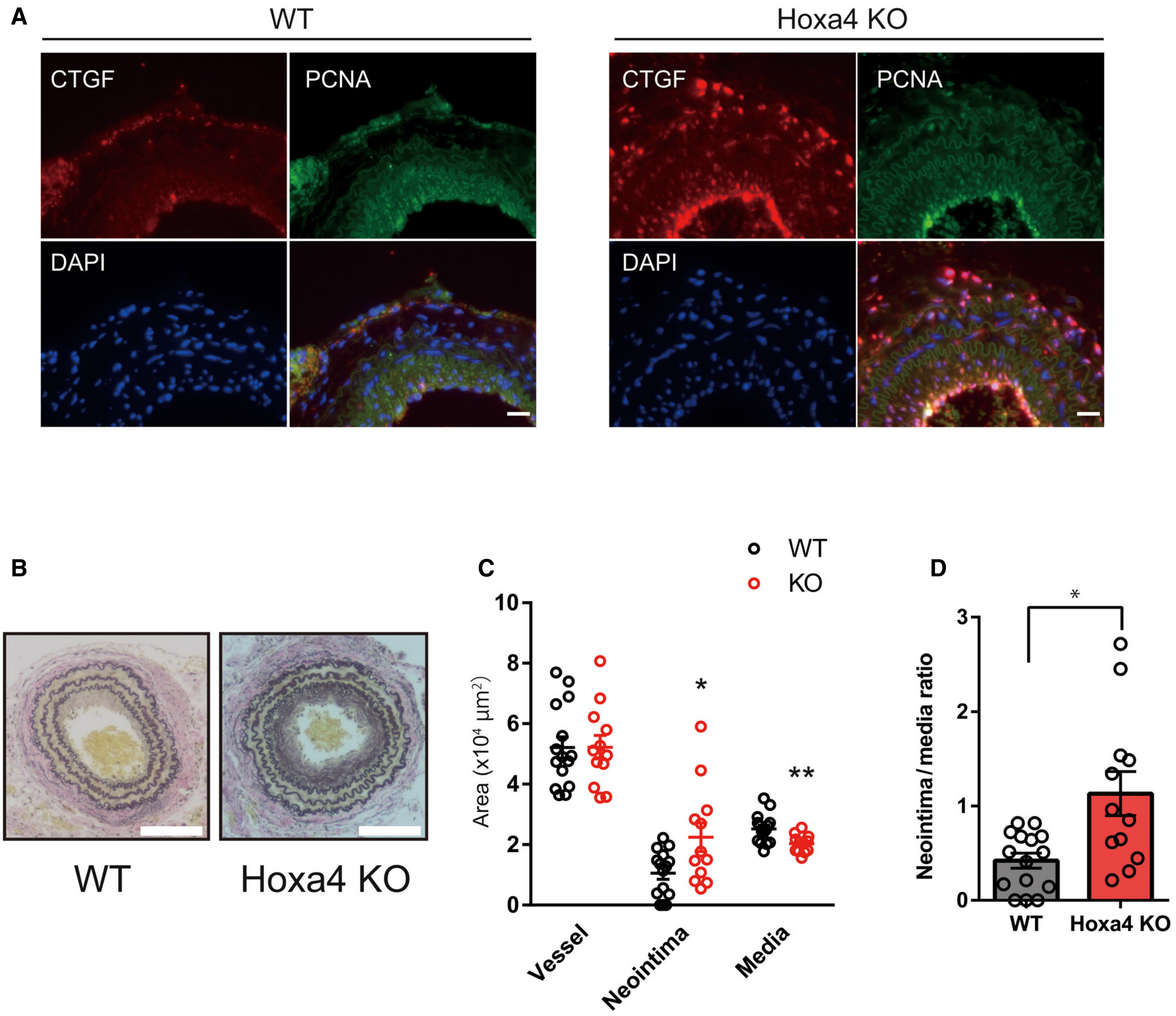

Figure EV5. Neointima formation in *Hoxa4*-deficient mice.

A Representative immunofluorescent staining images for CTGF and PCNA on the neointima in left carotid arteries at 4 weeks after ligation. Sections are obtained at 1,000 μm proximal to the ligation. Scale bars indicate 20 μm.

B Representative images showing EVG staining of left carotid arteries of WT and *Hoxa4* KO mice at 4 weeks after ligation. Sections are obtained at 500 μm proximal to the ligation. Scale bars indicate 100 μm.

C Vessel, neointima, and media area measured at 500 μm proximal to the ligation. Age- and sex-matched WT (n = 15) or *Hoxa4* KO (n = 12) mice were subjected to carotid artery ligation. $^*P < 0.05$, $^{**}P < 0.01$ versus WT, by unpaired two-tailed Mann–Whitney test. Data are presented as mean ± SEM.

D Neointima-to-media layer ratio measured at 500 μm proximal to the ligation. Age- and sex-matched WT (n = 15) or *Hoxa4* KO (n = 12) mice were subjected to carotid artery ligation. $^*P < 0.05$, by unpaired two-tailed Mann–Whitney test. Data are presented as mean ± SEM.

© 2020 The Authors
